# The Effect of Dietary Protein Hydrolysate from Black Soldier Fly Larvae and Schizochytrium on Palatability, Nutrient Metabolites and Health Status in Beagle Dogs

**DOI:** 10.3390/metabo14030165

**Published:** 2024-03-14

**Authors:** Yu Wei, Lingfeng Xue, Deying Ma, Yuxiao Weng, Mingkang Liu, Luyang Li, Ziyi Dai, Ziyun Zhao, Haifeng Wang, Xiao Xu

**Affiliations:** 1 Hubei Key Laboratory of Animal Nutrition and Feed Science, Wuhan Polytechnic University, Wuhan 430023, China; yuwei20000306@163.com (Y.W.); 15181488346@163.com (D.M.); 15527199297@163.com (M.L.); 17371453782@163.com (L.L.); dzy20022024@163.com (Z.D.); zzy15927868984@163.com (Z.Z.); 2 China Animal Husbandry Industry Co., Ltd., Beijing 100071, China; sworms@163.com; 3 P&O Biotechnology (Hubei) Co., Ltd., Wuhan 436043, China; 13707162068@139.com (Y.W.); 18627780422@126.com (H.W.)

**Keywords:** protein hydrolysate, black soldier fly larvae, schizochytrium, palatability, antioxidant, diarrhea, dogs

## Abstract

Protein hydrolysate from black soldier fly larvae (BSFP) has garnered great attention with its lower allergenicity, high amount of essential amino acids, and small bioactive peptides. Schizochytrium is a promising alternative source of n-3 FUFA because it has enriched docosahexaenoic acid (DHA, C22: 6). The aim of this study was to assess palatability, the presence of diarrhea, plasma biochemistry panels, anti-oxidative and anti-inflammatory effects, and immune function in beagle dogs when supplementing a mixture of protein hydrolysate from black soldier fly larvae and schizochytrium (BSFPs) into their diets. Experiment I: 24 young beagle dogs (16 males and 8 females; 4–5 months; BW: 6.40 ± 0.15 kg) were randomly divided into four groups: (1) control (CON), (2) 5% BSFPs, (3) 10% BSFPs, (4) 15% BSFPs. Their body weights and fecal scores were recorded, and blood samples were collected for analysis. Experiment II: three diets containing 5%, 10%, and 15% BSFPs were evaluated by comparing them with a basal diet (CON) to evaluate palatability. These results suggested that a lower presence of diarrhea existed in the BSFP diet than the CON diet (*p* < 0.05). Three treatment groups remarkably increased their total protein (TP) and albumin (ALB) contents and decreased their concentrations of triglyceride (TG) and total cholesterol (TC) in plasma (*p* < 0.05). Moreover, the 5% and 15% BSFPs groups had a higher calcium (CA) content in plasma, and the activities of aspartate aminotransferase (AST) and alanine aminotransferase (ALT) and contents of creatinine (CREA) and urea nitrogen (BUN) were significantly reduced by supplementing BSFP in their diets (*p* < 0.05). Their anti-oxidative enzyme activities of superoxide dismutase (SOD) and glutathione peroxidase (GSH-PX) were dramatically enhanced, and their malondialdehyde (MDA) concentrations were remarkably reduced (*p* < 0.05). Immunoglobulin A and G (IgA and IgG) concentrations in the plasma in the 10% and 15% BSFPs groups were significantly increased (*p* < 0.05). Furthermore, lower interleukin-8 (IL-8) contents were shown in the BSFP diets than the CON diet (*p* < 0.05). Similarly, the diets supplemented with BSFPs exhibited a positive effect on palatability (*p* < 0.05). To sum up, the diets supplemented with BSFPs significantly enhanced palatability, immune function, and anti-oxidative and anti-inflammatory capacity to alleviate diarrhea and improve the general health of the beagle dogs.

## 1. Introduction

Insect proteins are being increasingly considered as an alternative to chicken and fish meal in the commercial diet industry [1]. As a recent example, black soldier fly larvae (BSFL) have the huge potential to be a novel substitute in the market, especially in the pet market, because of their unique nutritional composition, including their high protein content, abundant amino acid composition, lauric acid, and essential minerals and vitamins [2,3]. Some studies have demonstrated that a BSFL-meal-supplemented diet had no negative influence on body health and was readily accepted by dogs [4], and the inclusion of BSFL in a cat diet was found to be more palatable and did not negatively affect fecal characteristics or blood biochemistry [5]. Moreover, hydrolysate showed more effective absorption and function due to a higher rate of pure protein and free amino acids and lower allergenicity compared with crude protein [6,7].

Recently, the synthesis of protein hydrolysate from black soldier fly larvae (BSFP) and its functional identification have received tremendous attention from the commercial diet industry. For example, BSFP has lower allergenicity and a high amount of essential amino acids, and some small bioactive peptides, such as anti-oxidative and immune ones, are produced after enzymatic hydrolyzing [8,9]. In addition, BSFP has a potent anti-oxidative capacity and can reduce reactive oxygen species (ROS) production and relieve oxidative stress in L-929 cells caused by H_2_O_2_ [10]. Moreover, the addition of 1% BSFP to their diet could improve growth performance and disease resistance, enhance anti-oxidative ability, and promote intestinal health and microbiota in largemouth bass [11].

Polyunsaturated fatty acids (PUFAs) are essential nutrients for animals to satisfy growth and maintain physiological function. For example, omega 3 (n-3) PUFAs have shown beneficial effects in immune function, such as inhibiting leucocyte chemotaxis, inflammatory cytokine production, and T-cell reactivity [12,13]. Marine algae such as a schizochytrium are a promising alternative source of n-3 FUFAs because they have enriched docosahexaenoic acid (DHA, C22: 6) and have been bioengineered to produce DHA for the food industry [14]. Early research has demonstrated that schizochytrium, as a new feed resource, could benefit animals and showed some positive effects when supplemented in the diet at 20 g/d schizochytrium, including an improved growth performance, anti-oxidative capacity, and feed efficiency in dairy calves before weaning [15]. When 0.5% schizochytrium was added to a poultry diet, the birds had enhanced egg production and quality and yolk DHA contents, and a decreased n-6/n-3 PUFA ratio [16]. Moreover, the positive influence of 0.4% schizochytrium supplementation in diets for dogs included increased palatability, protein digestibility, and oxidative stability [17]. For cats, the replacement of poultry fat with schizochytrium resulted in an improved inflammatory response and increased DHA deposition in their gonadal lipid profiles [18].

Based on current research, the additions of BSFP and schizochytrium have been found to improve domestic animal diets. However, there is limited research exploring the effect of supplementing both BSFP and schizochytrium (BSFPs) in the diets of domestic animals. Thus, the aim of this study was to determine the effects of diets supplemented with different concentrations of BSFP and schizochytrium on palatability, blood biochemical parameters, and antioxidant and anti-inflammatory properties in beagle dogs. This research may provide some information about the function of protein hydrolysate from black soldier fly larvae and schizochytrium in diets for companion animals, and the possibility of replacing chicken and fish meal and oil in the pet food market.

## 2. Materials and Methods

### 2.1. Experimental Animals and Design

All animal care and experimental procedures were approved by the Institutional Animal Care and Use Committee of Wuhan Polytechnic University (No. WPU202306065), and obeyed the Animal Scientific Procedures Act 1986 (Home Office Code of Practice. HMSO: London January 1997) and EU regulations (Directive 2010/63/EU).

#### 2.1.1. Experiment I: Feed Trial

Sixteen male and eight female healthy and young beagle dogs weighing 6.40 ± 0.15 kg and aged about 4–5 months were randomly divided into four treatments, with six dogs per treatment (four males and two females). Each dog was singly housed. The dietary treatments included the following: (1) a basal diet (CON group); (2) a basal diet that replaced chicken meal, fish meal, chicken oil, and fish oil with 5% of a mixture of protein hydrolysate from black soldier fly larvae and schizochytrium (5% BSFP group); (3) a basal diet that replaced chicken meal, fish meal, chicken oil, and fish oil with 10% of a mixture of protein hydrolysate from black soldier fly larvae and schizochytrium (10% BSFP group); and (4) a basal diet that replaced chicken meal, fish meal, chicken oil, and fish oil with 15% of a mixture of protein hydrolysate from black soldier fly larvae and schizochytrium (15% BSFP group). The mixture of protein hydrolysate from black soldier fly larvae and schizochytrium (BSFP) was a commercial product provided by P&O Biotechnology (Hubei) Co., Ltd., Ezhou, China. Diets were formulated to meet the nutrient needs of young dogs, as shown in Table 1, according to the Association of American Feed Control Officials [19]. During the experiment, all dogs were fed twice per day at 8:30 am and 16:30 pm, respectively, and provided with 80 g diets each time. Additionally, before the experiment, all dogs were treated with immunization and deworming, and free-choice water and a clean environment were provided for dogs. The trial lasted for 33 days, with the first 5 days as the adaptation period and 28 days as the trial period.

#### 2.1.2. Experiment II: Palatability Trial

Eighteen young Beagle dogs (the same four males and two females as the feed trial per treatment), aged about 4–5 months, weighing 6.40 ± 0.15 kg, were used to evaluate the palatability of the three treatment diets (5% BSFPs, 10% BSFPs, and 15% BSFPs). According to the international palatability comparison test method, the “Two-Bowl Test”, each palatability test was conducted for 2 days, in which the position of the bowl was changed on the second day, and we compared the CON group vs. 5% BSFP group, the CON group vs. 10% BSFP group, and the CON group vs. 15% BSFP group. Each experiment was conducted as a split-plate test, where two stainless steel bowls containing 40 g of the diet were presented to the dogs for 5 min. Preference was determined by calculating the intake ratio between the diets and by recording the first choice; the detailed operation followed the previous method with a slight change [20]. The “first sniff”, being the first food bowl the dog touched, and the “first bite”, being the first food bowl the dog took, were recorded separately, and then we calculated the relative ratio. The feed intake was calculated based on the amount of each diet consumed by each dog in 5 min.

First sniff (%) = number of Diet A/(total number of Diet A + Diet B)

First bite (%) = number of Diet A/(total number of Diet A + Diet B)

### 2.2. Sample Collection

On the first day of the trial period, all dogs were individually weighed and their initial body weights (IBWs) were recorded. On the 28th day, their final body weights (FBWs) were obtained. Fecal score (FS) was also evaluated throughout the whole experimental period considering a 5-point scale: 1 = hard, dry pellets, small hard mass; 2 = hard, formed, dry stool; 3 = soft, formed, and moist stool; 4 = soft, unformed stool, assumed shape of container; and 5 = watery, liquid that can be poured. A score of 1 ≤ FS < 2 was constipation, 2 ≤ FS < 3 was normal, 3 ≤ FS < 4 was soft stool, and 4 ≤ FS < 5 was diarrhea [21]. The diarrhea ratio was determined by recording the amount of diarrhea and total number of observations. After measuring the FBWs, blood samples were collected from the jugular vein and split between green-top plasma separator tubes and lavender-top ethylenediaminetetraacetic acid tubes. Green-top tubes were spun by centrifuging at 3500 rpm for 10 min, and plasma was extracted and stored at −80 °C for analysis. Lavender-top tubes were placed on a rocker and allowed to adequately mix with the anticoagulant.

### 2.3. Hematology Profile

The following hematological parameters, including white blood cells (WBCs), red blood cells (RBCs), hemoglobin (HB), lymphocytes (LYMs), and neutrophils (NEUs), were measured using a veterinary automatic hematology analyzer (BC-5000, MINDRAY, Shenzhen, China). According to previous research, the hematologic parameters were considerately assessed and the concrete reference interval was exhibited in the result [22].

### 2.4. Plasma Biochemical Parameters

The plasma chemistry analysis consisted of the following parameters: total protein (TP), albumin (ALB), glucose (GLU), triglyceride (TG), total cholesterol (TC), calcium (CA), phosphorus (P), aspartate aminotransferase (AST), alanine aminotransferase (ALT), creatinine (CREA), and urea nitrogen (BUN). These were determined by using an automatic biochemical analyzer (7100, HITACHI, Tokyo, Japan).

### 2.5. Antioxidative Capacity

According to the specification provided by the kit of Nanjing Jiancheng Institute of Biological Engineering (Nanjing, Jiangsu province, China), some antioxidative indicators, including the total antioxidant capacity (T-AOC), glutathione peroxidase (GSH-PX) and superoxide dismutase (SOD) enzyme activities, and malondialdehyde (MDA) content in the plasma, were evaluated. The absorbance value of the solution in the detection was taken by the Microplate Reader (168-1130 iMark, Bio-rad, Hercules, CA, USA).

### 2.6. Inflammatory Cytokines and Immune Levels

Some pro-inflammatory cytokines—tumor necrosis factor-α (TNF-α, range: 2.5–80 pg/mL, sensitivity < 0.1 pg/mL), interleukin 8 (IL-8, range: 7.5–240 pg/mL, sensitivity < 1 pg/mL), interleukin-1β (IL-1β, range: 20–640 pg/mL, sensitivity < 1 pg/mL)—and immunoglobulin contents, such as immunoglobulin A (IgA, range: 62.5–2000 ug/mL, sensitivity < 10 μg/mL), IgG (range: 0.5–16 g/L, sensitivity < 0.1 g/L), and IgM (range: 50–1600 μg/mL, sensitivity < 10 μg/mL) were measured by using canine enzyme-linked immunosorbent assay (ELISA) kits provided from Quanzhou Ruixin Biotechnology Co., Ltd., Fujiang, China.

### 2.7. Statistical Analyses

All data from the feed trial were analyzed using a MIXED procedure of SAS 9.1 software (SAS Inst. Inc., Cary, NC, USA), and the initial model included treatment, gender, and the interaction between treatment and gender as the main effects and replicates as random effects. However, no gender effects were significant. All data were presented as means and standard deviation (SD). When *p*-value < 0.05, the threshold for significance was reached, and individual means were compared by Duncan’s multiple-comparisons tests if significant effects were observed.

In the palatability trial, the first sniff, the first bite, and feed intake were all analyzed using a *t*-test after all data had been initially processed using Excel. Eighteen dogs in all were regarded as the experiment units for analysis.

## 3. Results

### 3.1. Body Weight, Stool Quality, and Diarrhea

A summary of the group mean final body weights, fecal scores, and the diarrhea ratios are presented in Table 2. No significant differences in the 28-day FBWs of the dogs were observed between the CON group and the diet treatments supplemented with BSFPs (*p* > 0.05). However, a significant difference in the fecal scores and the diarrhea ratios was presented in the diets: the diets supplemented with different levels of BSFPs significantly decreased the fecal scores and the diarrhea ratios compared with that of the CON group (*p* < 0.05). There was a dose-dependent relationship between the diarrhea rate and the content of BSFPs.

### 3.2. Hematology

As shown in Table 3, in comparison to the CON group, the supplementation of different levels of BSFPs in diets had no significant differences on white blood cells (WBCs), red blood cells (RBCs), hemoglobin (HB), lymphocytes (LYM), or neutrophils (NEUs) (*p* > 0.05). All hematology profiles remained with normal reference ranges throughout the trial.

### 3.3. Plasma Biochemistry Indicators

Compared with the CON group, there was a significant influence: the diet supplementation of 5%, 10%, and 15% BSFPs groups significantly increased the TP and ALB contents in the plasma of the dogs, as shown in Table 4 (*p* < 0.05). In addition, the contents of TG and TC were significantly decreased by the supplementation of different levels of BSFPs in diets compared to the CON group, and the addition of 5% BSFPs and 15% BSFPs in diets significantly increased the CA concentration in plasma (*p* < 0.05). Meanwhile, the contents in plasma of AST, ALT, CERA, and BUN were significantly reduced through supplementation with different levels of BSFPs in the diets compared to when fed with the CON diet (*p* < 0.05).

### 3.4. Anti-Oxidative Capacity

Table 5 shows that diet groups supplemented with different levels of BSFPs had increased GSH-PX activity in the plasma and the 5% BSFP and 15% BSFP groups exhibited significantly higher SOD activities than the CON group (*p* < 0.05). Moreover, the concentration of MDA in plasma was significantly reduced by feeding the dogs diets with inclusion of different levels of BSFPs compared with the CON diet (*p* < 0.05). Nevertheless, there was no difference in the T-AOC activity among dietary treatments (*p* > 0.05).

### 3.5. Inflammatory Cytokines and Immune Levels

Dogs fed diets supplemented with different levels of BSFPs had significantly decreased contents of IL-8 in plasma compared to those fed the CON diet, as shown in Table 6 (*p* < 0.05). However, no significant differences were observed in the contents of TNF-α and IL-1β among dietary treatments (*p* > 0.05). The concentrations of IgA and IgG were significantly higher in dogs whose diets were supplemented with 10% and 15% BSFPs than the CON diet and 5% BSFP diet, as shown in Table 7 (*p* < 0.05). However, the dietary treatments did not affect the plasma concentration of IgM (*p* > 0.05).

### 3.6. Palatability

Shown in Table 8 are the results of the palatability test of the diets supplemented with different levels of BSFPs (5%, 10%, and 15% BSFPs groups) and the basal diet (CON group). The significant effects observed were that dogs fed the diets composed of different levels of BSFPs exhibited more positive results on the first sniff, the first bite, and feed intake than those on the basal diet (*p* < 0.05).

## 4. Discussion

As our results described, the diets supplemented with a mixture of protein hydrolysate from black soldier fly larvae and schizochytrium (BSFPs) to replace partial fish meal, chicken meal, fish oil, and chicken oil had no negative influence on final body weight. Consistent with our study, no adverse effects occurred in body weight by feeding adults dogs diets supplemented with up to 20% BSFL meal and up to 5% BSFL oil [4]. In addition, compared with the CON diet, diets with added levels of 5%, 10%, and 15% BSFPs had significantly improved fecal scores and decreased ratios of diarrhea, and 15% BSFPs was more efficient among the treatments. The dietary treatments involving the inclusion of BSFL meal, whole BSFL, and BSFL oil displayed a significant tendency to improve fecal score for cats [5], and having 4% and 8% BSFL supplemented in their diets significantly reduced the incidences of diarrhea induced by enterotoxigenic *Escherichia coli K88* in weaned piglets [23]. In addition, an early study found that DHA added to infant formula decreased infant diarrhea [24], and the dietary intake schizochytrium, rich in DHA, showed the same effect: when supplemented at 20 g/d, schizochytrium significantly reduced the diarrhea frequency caused by *Escherichia coli K99* in pre-weaning dairy calves [25]. Based on these studies, our results are the same as our expectation that a mixture of BSFPs would have a similar effect on alleviating diarrhea.

Hematology profiles (WBCs, RBCs, HB, LYMs, and NEUs), indicative of the dogs’ health, were maintained within the normal range and indicated that the diets supplemented with BSFPs did not adversely affect any health outcomes. These results were similar to those of previous research with BSFL meal and oil for cats and dogs [26,27], for fish [28], and in poultry [29,30]. Likewise, there was no negative influence on hematology profiles from the addition of 0.4% schizochytrium in the diet of dogs [17] or diets supplemented with schizochytrium at 170 g/d and 255 g/d for dairy cows [31]. The blood biochemistry parameters are a crucial indicator of health and may fluctuate due to a variety of factors such as the environment, disease, and nutrition. In this study, the dietary treatments including different levels of BSFPs significantly enhanced the contents of TP, ALB, and CA, and decreased the TG and TC concentrations in plasma. Similar findings were found in previous research, which indicated that the supplementation of 2.5% and 25% BSFL meal could improve the TP contents, respectively, in Brahma chickens and Clarias gariepinus [32,33], and diets that included 75% and 100% BSFL oil to replace soybean oil could significantly increase the ALB content in plasma [34]. A possible reason for this consequence is that protein hydrolysate from black soldier fly larvae (BSFP) is easier for animals’ intestines to absorb than crude protein due to a larger percentage of pure protein and free amino acids, as well as a high protein efficiency ratio [7,8]. Additionally, the substitution of fish meal with BSFL meal in the diet resulted in a lower TC and TG concentration in juvenile striped catfish [35], which could be responsible for the presence of chitin in BSFL, which plays an important role in triglyceride hydrolysis and in boosting lipid utilization [36]. The low-level dietary fishmeal supplemented with 0.3% and 0.6% schizochytrium also significantly reduced the content of TG and TC [37] and had the potential to increase the CA concentration in plasma [16]. Levels of enzyme activities, such as AST and ALT, and contents of CREA and BUN are related to the health of the liver and kidney. Similar to our results, the addition of BSFL meal led to a reduction in AST and ALT for African catfish [33]. Moreover, many studies have demonstrated that DHA also reduces high-fat-diet induced AST and ALT contents [38] and alleviates bisphenol (BPA)-induced nephrotoxicity by decreasing BUN and CERA concentrations in plasma [39].

Oxidative stress is caused by an imbalance between the oxidative and anti-oxidative systems, which can produce excessive reactive oxygen species (ROS) and damage to DNA and tissue [40]. The enzyme activities of T-AOC, GSH-PX, and SOD are essential components associated with the antioxidant defense system, protecting cells from ROS-induced oxidative damage, and MDA is an important marker of oxidative status generated by ROS attacking PUFAs in membrane phospholipids [41]. In our experiment, the supplementation of different levels of BSFPs in diets significantly promoted the activity of GSH-PX and SOD and reduced the MDA content in plasma for dogs. Consistent with our results, schizochytrium exhibited a positive influence on anti-oxidation, which promoted the activity of GSH-PX in beef, the activity of SOD in juvenile mirror carp, and the reduction in MDA content in largemouth bass [42,43,44]. Moreover, early studies have verified that protein hydrolysate from black soldier fly larvae (BSFP) has potent antioxidant activity [9,10]. As we expected, BSFPs also have potential antioxidant capacity.

Plasma immunoglobulins such as IgA, IgM, and IgG are usually used to evaluate an animal’s immune system and its capacity to detect pathogenic invasion. Our research showed that the inclusion of 10% and 15% BSFPs in diets caused higher contents of IgA and IgG in plasma for dogs. Previous studies have shown that microalgae in animal feed could activate the immune system, and a diet with added spirulina boosted the immunoglobulin concentration and immunological responses [45]. In addition, the replacement of soybean meal with 25% BSFL meal also enhanced immune levels by increasing IgA, IgG, and IgM in the ileum of piglets [46], which was attributed to the lauric acid found in BSFL that might increase immunoglobulin synthesis by reducing interleukin production [47]. Another reason was the chitooligosaccharides produced by the chitin in BSFL were able to promote immune responses according to the report [48]. An amount of production of pro-inflammatory cytokines such as TNF-α, IL-1β, and IL-8 are relevant to tissue injuries, sepsis, and noninfectious systemic inflammatory response syndrome in dogs [49]. The dietary supplementation of 5%, 10%, and 15% BSFPs could significantly reduce the concentration of IL-8 in the plasma of dogs. Research have shown that the anti-inflammatory activity of BSFL oil alleviated dextran sulfate sodium (DSS)-induced colitis by modulating Toll-like receptor (TLR) signaling [50], and the antimicrobial peptides (AMPs) from BSFL reduced LPS-induced nitric oxide and cytokine production in murine macrophage cells [51]. Furthermore, protein hydrolysate from black soldier fly larvae (BSFP), like other enzymatically hydrolyzed proteins from edible insects, might have anti-inflammatory capacity [52]. In the same way, the dietary addition of 15% schizochytrium powder could maintain a normal physiological state in the intestine by downregulating the gene expression of *IL-6*, *IL-8*, and *IL-1β* [53], and the schizochytrium oil also significantly reduced the concentration of pro-inflammatory cytokines, TNF-α, IL-6, and IL-1β, in mice treated with ceftriaxone sodium [54]. In addition, the supplementation of goats’ diet with 20 g/d schizochytrium resulted in a downregulation of pro-inflammatory transcription by modifying TLR4 regulation in monocytes and neutrophils [55].

Palatability is the major criterion generally used to evaluate product performance in the pet food market and is linked with pleasure perception or liking during consumption and seen as readily accepted by animals in brief [56,57]. The inclusion of different levels of BSFPs in the diets positively influenced the first sniff, the first bite, and the feed intake for dogs, especially the addition of 15% BSFPs in the diet, suggesting that BSFP is a palatable ingredient. The most likely reasons for this are that the particular flavor of microalgae schizochytrium, the potential flavor of Maillard during protein hydrolysates [58], and the fact the proteins are broken into short peptides so that the protein hydrolysates can not be recognized by the immune system, thus reducing the allergenicity [7]. Similar to the present study, the addition of 0.4% of schizochytrium to the diet was palatable to dogs due to the enhanced concentrations of fatty acids [17], and the dietary inclusion of BSFL positively influenced palatability through enhancing food consumption, and made it the first-choice preference by cats [5].

## 5. Conclusions

Overall, the diet supplemented with different levels of a mixture of protein hydrolysate from black soldier fly larvae and schizochytrium (BSFPs) significantly enhanced the palatability, immune level, anti-oxidative and anti-inflammatory capacity to alleviate diarrhea and body health in beagle dogs. In particular, 15% BSFPs have a more efficient influence on palatability, anti-oxidation, and anti-inflammation.

## Figures and Tables

**Table 1:** Ingredient composition of the dogs’ diets containing graded inclusion levels of a mixture of protein hydrolysate from black soldier fly larvae and schizochytrium (BSFPs).

Ingredients, %	CON	5% BSFPs	10% BSFPs	15% BSFPs
Protein hydrolysate from black soldier fly larvae and schizochytrium	0	5.00	10.00	15.00
Chicken meal	45.50	42.00	38.50	35.00
Fish meal	6.50	6.00	5.50	5.00
Chicken liver meal	2.00	2.00	2.00	2.00
Chicken oil	7.00	6.50	6.00	5.50
Fish oil	2.50	2.00	1.50	1.00
Powdered egg yolk	2.50	2.50	2.50	2.50
Sweet potato granule	20.00	20.00	20.00	20.00
Pumpkin powder	4.00	4.00	4.00	4.00
Carrot powder	4.00	4.00	4.00	4.00
Distillers yeast	1.00	1.00	1.00	1.00
Flaxseed	1.00	1.00	1.00	1.00
Kelp powder	1.00	1.00	1.00	1.00
Cranberry	1.00	1.00	1.00	1.00
Beet pulp	0.50	0.50	0.50	0.50
Premix ^1^	1.50	1.50	1.50	1.50
Total	100.00	100.00	100.00	100.00
Nutrient level ^2^				
ME, kcal/kg	3880	3975	3950	3965
CP, %	19.50	19.50	19.50	19.50
EE, %	14.60	14.50	14.50	14.40
Ca, %	0.60	0.62	0.64	0.65
P, %	0.50	0.51	0.51	0.52

CON: basal diet group; 5% BSFPs; group consuming a basal diet supplemented with 5% of protein hydrolysate from black soldier fly larvae and schizochytrium; 10% BSFP, group consuming a basal diet supplemented with 10% of protein hydrolysate from black soldier fly larvae and schizochytrium; 15% BSFP, group consuming a basal diet supplemented with 15% of protein hydrolysate from black soldier fly larvae and schizochytrium; ME: metabolic energy; CP: crude protein; EE: ether extract; Ca: calcium; P: phosphorus. ^1^ Provided per kg diet: Cu (CuSO_4_), 15 mg; K (KIO_3_), 10 mg; Fe (C_6_H_5_FeO_7_), 100 mg; Mn (MnCO_3_), 25 mg; Na (Na_2_SeO_3_), 154.0 mg; Zn (ZnSO_4_), 145 mg; Co (CoSO_4_), 1.5 mg; vitamin A, 20,000 IU; vitamin D3, 2500 IU; vitamin E, 45 mg; vitamin K3, 5 mg; vitamin B12, 0.03 mg; biotin, 0.07 mg; folic acid, 0.4 mg; nicotinic acid, 55 mg; vitamin B5, 16.80 mg; pyridoxine, 10.2 mg; riboflavin, 10 mg; thiamin, 8.5 mg; choline, 750 mg. ^2^ The nutrient levels were calculated values.

**Table 2:** Effects of protein hydrolysate from black soldier fly larvae and schizochytrium (BSFPs) on the body weight, stool quality, and diarrhea of beagle dogs.

Item	CON	5% BSFPs	10% BSFPs	15% BSFPs	*p*-Value
IWB, kg	6.37 ± 0.18	6.38 ± 0.14	6.37 ± 0.15	6.38 ± 0.16	0.859
FWB, kg	6.71 ± 0.22	6.79 ± 0.21	6.69 ± 0.25	6.95 ± 0.20	0.635
Fecal score	2.85 ± 0.09 ^a^	2.62 ± 0.08 ^b^	2.52 ± 0.09 ^b^	2.50 ± 0.09 ^b^	<0.05
Diarrhea, %	4.76 ± 0.18 ^a^	2.82 ± 0.15 ^b^	1.85 ± 0.14 ^c^	0.52 ± 0.11 ^d^	<0.01

N = 6 (1 dog per cage). IWB, initial body weight; FWB, final body weight; CON, fed with basal diet; 5% BSFPs; group fed a basal diet with chicken meal, fish meal, chicken oil, and fish oil replaced with 5% of protein hydrolysate from black soldier fly larvae and schizochytrium; 10% BSFP, group fed a basal diet with chicken meal, fish meal, chicken oil, and fish oil replaced with 10% of protein hydrolysate from black soldier fly larvae and schizochytrium; 15% BSFP, group fed a basal diet with chicken meal, fish meal, chicken oil, and fish oil replaced with 15% of protein hydrolysate from black soldier fly larvae and schizochytrium. ^a–d^ Different letters on the shoulder indicate significant differences between mean values for a given behavior (*p* < 0.05).

**Table 3:** Effects of protein hydrolysate from black soldier fly larvae and schizochytrium (BSFPs) on hematology profile of beagle dogs.

Item	Reference Interval	CON	5% BSFPs	10% BSFPs	15% BSFPs	*p*-Value
WBC, 10^9^/L	5.84–20.26	11.43 ± 2.24	10.68 ± 2.20	9.89 ± 2.18	14.66 ± 2.32	0.385
RBC,10^12^/L	5.58–9.08	6.03 ± 0.65	5.56 ± 0.62	5.75 ± 0.66	5.63 ± 0.63	0.716
HB, g/L	120–180	133 ± 11	123 ± 12	119 ± 12	126 ± 14	0.869
LYM, 10^9^/L	2.04–4.66	3.33 ± 0.60	3.27 ± 0.55	3.69 ± 0.54	3.21 ± 0.53	0.302
NEU, 10^9^/L	4.27–9.06	7.45 ± 0.65	5.73 ± 0.68	5.50 ± 0.63	8.42 ± 0.68	0.580

N = 6 (1 dog per cage). WBC, white blood cell; RBC, red blood cell; HB, hemoglobin; LYM, hemoglobin; NEU, neutrophil.

**Table 4:** Effects of protein hydrolysate from black soldier fly larvae and schizochytrium (BSFPs) on plasma biochemistry indicators of beagle dogs.

Item	CON	5% BSFPs	10% BSFPs	15% BSFPs	*p*-Value
TP, g/L	46.35 ± 1.12 ^b^	58.39 ± 1.20 ^a^	57.36 ± 1.18 ^a^	60.21 ± 1.25 ^a^	<0.05
ALB, g/L	14.85 ± 1.00 ^c^	20.54 ± 1.08 ^b^	24.80 ± 1.10 ^a^	24.32 ± 1.10 ^a^	<0.05
GLU, mmol/L	3.45 ± 0.49	4.28 ± 0.47	4.22 ± 0.46	4.56 ± 0.47	0.102
TG, mmol/L	0.58 ± 0.09 ^a^	0.32 ± 0.06 ^b^	0.36 ± 0.07 ^b^	0.36 ± 0.07 ^b^	<0.05
TC, mmol/L	4.02 ± 0.13 ^a^	2.25 ± 0.11 ^b^	2.41 ± 0.09 ^b^	2.21 ± 0.10 ^b^	<0.05
CA, mmol/L	2.25 ± 0.11 ^b^	2.65 ± 0.13 ^a^	2.45 ± 0.13 ^ab^	2.78 ± 0.15 ^a^	<0.05
P, mmol/L	1.85 ± 0.11	1.74 ± 0.10	1.88 ± 0.14	1.72 ± 0.15	0.884
AST, U/L	33.95 ± 3.32 ^a^	20.35 ± 2.95 ^b^	14.71 ± 2.21 ^bc^	12.66 ± 2.33 ^c^	<0.01
ALT, U/L	21.36 ± 2.62 ^a^	18.32 ± 2.45 ^b^	15.92 ± 2.43 ^b^	13.13 ± 2.50 ^b^	<0.01
CREA, umol/L	85.32 ± 2.17 ^a^	50.66 ± 1.95 ^b^	48.32 ± 1.90 ^b^	44.21 ± 1.88 ^b^	<0.05
BUN, mmol/L	12.55 ± 0.22 ^a^	8.63 ± 0.16 ^b^	7.99 ± 0.15 ^c^	7.62 ± 0.14 ^c^	<0.05

N = 6 (1 dog per cage). TP, total protein; ALB, albumin; GLU, glucose; TG, triglyceride; TC, total cholesterol; CA, calcium; P, phosphorus; AST, aspartate aminotransferase; ALT, alanine aminotransferase; CREA, creatinine; BUN, urea nitrogen. ^a–c^ Different letters on the right indicate significant differences between the mean values for a given behavior (*p* < 0.05).

**Table 5:** Effects of protein hydrolysate from black soldier fly larvae and schizochytrium (BSFPs) on plasma anti-oxidative level of beagle dogs.

Item	CON	5% BSFPs	10% BSFPs	15% BSFPs	*p*-Value
T-AOC, mM	1.07 ± 0.06	1.10 ± 0.07	1.16 ± 0.07	1.15 ± 0.07	0.452
GSH-PX, U/mL	965 ± 93 ^b^	1321 ± 131 ^a^	1457 ± 147 ^a^	1486 ± 141 ^a^	<0.05
SOD, U/mL	42.63 ± 3.11 ^b^	52.40 ± 3.75 ^a^	50.86 ± 3.62 ^ab^	55.91 ± 3.51 ^a^	<0.05
MDA, nmol/mL	2.97 ± 0.18 ^a^	2.13 ± 0.15 ^b^	2.03 ± 0.13 ^b^	2.19 ± 0.16 ^b^	<0.05

N = 6 (1 dog per cage). T-AOC, total antioxidant capacity; GSH-PX, glutathione peroxidase; SOD, superoxide dismutase; MDA, malondialdehyde. ^a–b^ Different letters on the shoulder indicate significant differences between mean values for a given behavior (*p* < 0.05).

**Table 6:** Effects of protein hydrolysate from black soldier fly larvae and schizochytrium (BSFPs) on pro-inflammatory cytokine levels of beagle dogs.

Item	CON	5% BSFPs	10% BSFPs	15% BSFPs	*p*-Value
TNF-α, pg/mL	24.44 ± 2.73	22.48 ± 2.25	25.85 ± 2.31	25.02 ± 2.60	0.873
IL-1β, pg/mL	47.44 ± 4.84	44.80 ± 4.55	45.76 ± 4.93	53.58 ± 5.24	0.662
IL-8, pg/mL	62.01 ± 2.77 ^a^	29.81 ± 1.83 ^c^	41.21 ± 1.95 ^b^	27.24 ± 1.72 ^c^	<0.05

N = 6 (1 dog per cage). TNF-α, tumor necrosis factor-α; IL-1β, interleukin-1β; IL-8, interleukin 8. ^a–c^ Different letters on the right indicate significant differences between mean values for a given behavior (*p* < 0.05).

**Table 7:** Effects of protein hydrolysate from black soldier fly larvae and schizochytrium (BSFPs) on immunoglobulin levels of beagle dogs.

Item	CON	5% BSFPs	10% BSFPs	15% BSFPs	*p*-Value
IgA, ug/mL	421 ± 41 ^b^	486 ± 43 ^b^	580 ± 47 ^a^	587 ± 50 ^a^	<0.05
IgG, ug/mL	1238 ± 113 ^b^	1432 ± 132 ^b^	3257 ± 225 ^a^	3710 ± 267 ^a^	<0.05
IgM, ug/mL	241 ± 27	204 ± 23	253 ± 31	265 ± 35	0.186

N = 6 (1 dog per cage). IgA, immunoglobulin A; IgG, immunoglobulin G; IgM, immunoglobulin M. ^a–b^ Different letters on the right indicate significant differences between mean values for a given behavior (*p* < 0.05).

**Table 8:** Effects of protein hydrolysate from black soldier fly larvae and schizochytrium (BSFPs) on palatability of beagle dogs.

Item	CON	5% BSFPs	*p*-Value
First sniff, %	46.88 ± 2.45	53.12 ± 2.57	0.040
First bite, %	47.50 ± 2.31	52.50 ± 2.37	0.061
Feed intake, g/5 min	33.37 ± 1.88	37.98 ± 1.85	0.038
	**CON**	**10% BSFPs**	
First sniff, %	48.44 ± 2.02	51.56 ± 2.13	0.078
First bite, %	41.67 ± 2.23	58.33 ± 2.43	<0.001
Feed intake, g/5 min	30.75 ± 1.92	36.48 ± 1.98	<0.001
	**CON**	**15% BSFPs**	
First sniff, %	45.00 ± 2.84	55.00 ± 2.91	<0.001
First bite, %	46.52 ± 2.25	53.75 ± 2.46	<0.001
Feed intake, g/5 min	32.98 ± 2.00	38.85 ± 2.08	<0.001

N = 6 (1 dog per cage). Significant differences existed between CON group and treatments (5% BSFPs, 10% BSFPs and 15% BSFPs) at *p*-value < 0.05.

## Data Availability

All data are shown in the manuscript.
